# The blue light-induced interaction of cryptochrome 1 with COP1 requires SPA proteins during Arabidopsis light signaling

**DOI:** 10.1371/journal.pgen.1007044

**Published:** 2017-10-09

**Authors:** Xu Holtkotte, Jathish Ponnu, Margaret Ahmad, Ute Hoecker

**Affiliations:** 1 Botanical Institute and Cluster of Excellence on Plant Sciences (CEPLAS), Biocenter, University of Cologne, Cologne, Germany; 2 UMR 8256 (B2A) CNRA—UPMC, IBPS, Université Pierre et Marie Curie, 9 quai Saint Bernard, Paris, France; University of Lausanne, SWITZERLAND

## Abstract

Plants constantly adjust their growth, development and metabolism to the ambient light environment. Blue light is sensed by the Arabidopsis photoreceptors CRY1 and CRY2 which subsequently initiate light signal transduction by repressing the COP1/SPA E3 ubiquitin ligase. While the interaction between cryptochromes and SPA is blue light-dependent, it was proposed that CRY1 interacts with COP1 constitutively, i.e. also in darkness. Here, our *in vivo* co-immunoprecipitation experiments suggest that CRY1 and CRY2 form a complex with COP1 only after seedlings were exposed to blue light. No association between COP1 and CRY1 or CRY2 was observed in dark-grown seedlings. Thus, our results suggest that cryptochromes bind the COP1/SPA complex after photoactivation by blue light. In a *spa* quadruple mutant that is devoid of all four SPA proteins, CRY1 and COP1 did not interact *in vivo*, neither in dark-grown nor in blue light-grown seedlings. Hence, SPA proteins are required for the high-affinity interaction between CRY1 and COP1 in blue light. Yeast three-hybrid experiments also show that SPA1 enhances the CRY1-COP1 interaction. The coiled-coil domain of SPA1 which is responsible for COP1-binding was necessary to mediate a CRY1-SPA1 interaction *in vivo*, implying that—in turn—COP1 may be necessary for a CRY1-SPA1 complex formation. Hence, SPA1 and COP1 may act cooperatively in recognizing and binding photoactivated CRY1. In contrast, the blue light-induced association between CRY2 and COP1 was not dependent on SPA proteins *in vivo*. Similarly, ΔCC-SPA1 interacted with CRY2, though with a much lower affinity than wild-type SPA1. In total, our results demonstrate that CRY1 and CRY2 strongly differ in their blue light-induced interaction with the COP1/SPA complex.

## Author summary

Plants sense ambient light conditions through several photoreceptors that induce a complex signaling cascade to vastly alter gene expression, growth and development. Blue light activates two cryptochrome photoreceptors which subsequently inactivate an E3 ubiquitin ligase consisting of COP1 and SPA proteins. While COP1 also exists in humans where it polyubiquitinates a number of proteins, e.g. p53, in a light-independent fashion, it is known that SPA proteins are specific to the green lineage. Here, we show that SPA proteins are required for the blue light-induced interaction between cryptochrome 1 and COP1. We thus provide mechanistic evidence that SPA proteins may have evolved in plants to place the activity of the COP1 E3 ubiquitin ligase under the control of light.

## Introduction

Plants constantly adjust their growth and development to the ambient light environment. To sense light, plants have evolved several sets of photoreceptors which include the blue light-absorbing cryptochromes, phototropins and the ZEITLUPE family, the red/far-red responsive phytochromes and the UV-B photoreceptor UVR8. These photoreceptors regulate growth and development throughout the plant life cycle, such as seed germination, seedling deetiolation, shade avoidance and the induction of flowering. Hence, light signaling is strongly interconnected with many endogenous developmental pathways [1, 2].

There are three Arabidopsis cryptochromes—CRY1, CRY2 and CRY3—of which the nuclear-localized CRY1 and CRY2 are well characterized as the primary cryptochrome blue light (B) receptors involved in light-controlled plant development. CRY3, in contrast, is a DASH-type cryptochrome that is localized in chloroplasts and mitochondria. It retains DNA repair activity but its role in signaling is not understood [3]. CRY1 and CRY2 have partially overlapping but also distinct functions in photomorphogenesis. CRY1 primarily functions in B-induced seedling deetiolation, while CRY2 is mainly responsible for the induction of flowering under long day photoperiods, though it also contributes to seedling deetiolation under low fluence rates of B. CRY1 and CRY2 also differ in their stability: CRY1 is a relatively stable photoreceptor, while CRY2 is rapidly degraded in B via the ubiquitin-proteasome pathway. The CRY1 and CRY2 photoreceptors carry two chromophores that are responsible for light absorption, the primary chromophore FAD and a pterin as a second chromophore. These chromophores are attached to the highly conserved N-terminal domain of the cryptochrome apoproteins, the Photolyase Homologous Region (PHR). This domain is also responsible for homodimerization of CRY1 and CRY2. The CRY C-terminal Extensions (CCE) in CRY1 and CRY2 are only loosely conserved but important for cryptochrome signaling activities [4–6].

Upon blue light absorption, CRY2 homodimerizes and both CRY1 and CRY2 are phosphorylated at multiple residues which is associated with cryptochrome signaling activity [7–10]. Recently, a kinase responsible for CRY2 phosphorylation was identified [11]. Photoactivated cryptochromes induce signaling via multiple mechanisms [5, 12, 13]. A key mechanism is based on the B-induced interaction of CRY1 and CRY2 with SUPPRESSOR OF PHYA-105 (SPA) proteins which leads to an inactivation of the CONSTITUTIVELY PHOTOMORPHOGENIC1 (COP1)/SPA E3 ubiquitin ligase [14–16].

In dark-grown plants, the COP1/SPA complex suppresses light signaling by polyubiquitinating positive regulators of light signaling, mainly transcription factors, which results in their degradation in the 26S proteasome. COP1/SPA is the substrate-recognition unit of a CULLIN4 (CUL4)-based multi-subunit E3 ubiquitin ligase. It is a tetramer of two COP1 and two SPA proteins of the four-member SPA protein family [17, 18]. Genetic analysis indicates that both COP1 and SPA proteins are necessary for activity of this E3 ligase [19–21]. When plants are exposed to light, cryptochrome and phytochrome photoreceptors interact with SPA1, thereby inhibiting the COP1/SPA complex which causes a stabilization of the substrate transcription factors and subsequent photomorphogenesis [17, 18]. At least three mechanisms inactivate COP1/SPA in the light. COP1 is shuttled from the nucleus into the cytosol upon light exposure of seedlings [22, 23]. Light causes a dissociation of the COP1/SPA interaction [15, 16, 24, 25]. Light causes a destabilization of SPA1 and, in particular, SPA2, in a fashion that is specific to phytochrome photoreceptors [26, 27].

CRY1 and CRY2 differ in that only the light-labile CRY2 is destabilized in B, which is partly due to the COP1/SPA E3 ligase [7, 28]. CRY1 and CRY2 also differ in their interactions with SPA proteins. The CCE domain of CRY1 interacts with the WD-repeat domain of SPA1, whereas the PHR domain of CRY2 interacts with the N-terminal kinase-like domain of SPA1. This suggests that CRY1 and CRY2 act differently in the inactivation of COP1/SPA in B. Indeed, only CRY1 causes a dissociation of COP1/SPA in B, while the B-dependent association between CRY2 and SPA1 promotes the binding of CRY2 to COP1 [14–16]. Like SPA1, COP1 also directly interacts with CRY1 and CRY2 [29, 30], but—unlike SPA1—it was suggested in early studies that COP1 interacts with CRY1 irrespective of light conditions, i.e. in both dark- and light-grown seedlings [30].

The exact role of SPA proteins in the COP1/SPA complex is so far not understood. Since COP1 in humans acts without SPA proteins, which are specific to the green lineage, it was suggested that SPA proteins might have evolved to place the activity of COP1/SPA under the control of light. Indeed, recent evidence shows that SPA proteins are required for the light-induced relocalization of COP1 from the nucleus to the cytosol [31]. Here, we have analyzed the interaction of COP1 with cryptochromes in the presence and absence of SPA proteins, i.e. in a *SPA* wild-type and in a *spa* quadruple mutant background. We show that in a *SPA* wild-type, COP1 associates with cryptochromes only in B-exposed seedlings, indicating that COP1, like SPA1, interacts with CRY1 and CRY2 only in B. We further demonstrate that SPA proteins are required for the B-induced interaction of CRY1 with COP1 *in vivo*. CRY2, in contrast, is capable of interacting with COP1 in a SPA-independent manner *in vivo*.

## Results

### Exposure to B is required for an *in vivo*-association of CRY1 and CRY2 with COP1

**Fig 1:**
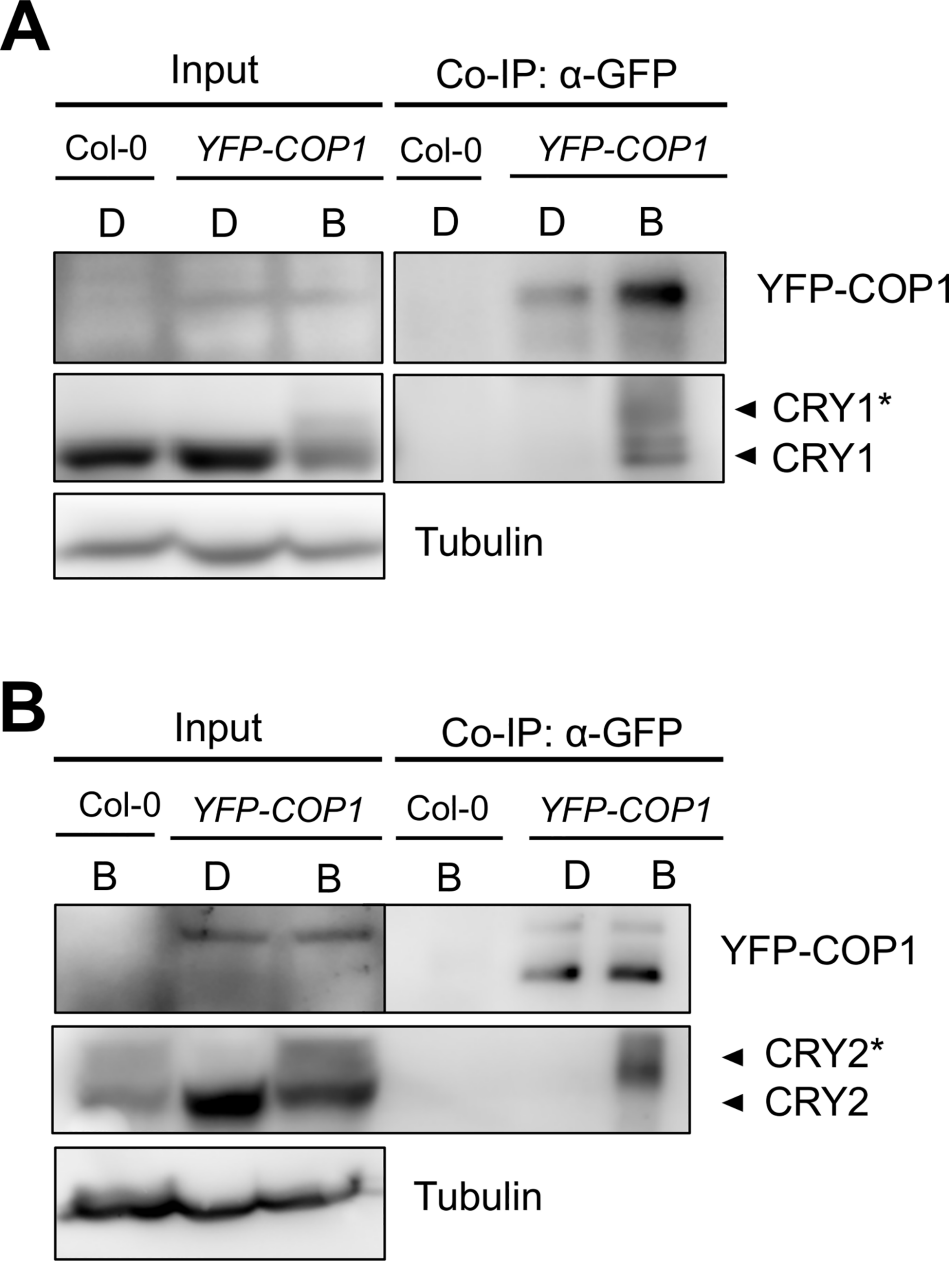
COP1 associates with CRY1 and CRY2 in a blue-light dependent manner *in vivo*. (**A**, **B**) Co-immunoprecipitation of CRY1 (**A**) and CRY2 (**B**) by YFP-COP1. Transgenic 35S::*YFP-COP1* seedlings were grown in darkness (D) for 4 days and subsequently transferred to blue light (B) of a fluence rate of 50 μmol m^-2^ s^-1^ for 1 h (**A**) or 5 min (**B**). Protein extracts were immunoprecipitated using α-GFP beads. YFP-COP1 was detected using α-GFP antibodies; CRY1 and CRY2 were detected using α-CRY1 and α-CRY2 antibodies. Asterisks likely indicate phosphorylated CRY1 and CRY2, respectively. All signals in **(A)** and **(B)** were from the same respective membrane. The YFP-COP1 signals **(A, B)** of the input samples were from longer exposures than those from the co-immunoprecipitates. The CRY1 signals **(A)** of the input samples were from shorter exposure than those from the co-immunoprecipitates.

To investigate whether cryptochromes associate with COP1 in a B-dependent manner *in planta*, we carried out co-immunoprecipitation studies using transgenic Arabidopsis seedlings expressing YFP-COP1 (*35S*::*YFP-COP1*) [32]. Both CRY1 and CRY2 were co-immunoprecipitated by YFP-COP1 only in B-exposed and not in dark-grown seedlings (Fig 1A and 1B; S1A Fig), indicating that the association between cryptochromes and COP1 is B-dependent. Both CRY1 and CRY2 undergo phosphorylation in B and the phosphorylated isoforms of cryptochromes have been proposed to be physiologically active [7, 8, 11]. Indeed, YFP-COP1 preferentially bound to higher-mobility isoforms of CRY1 and CRY2 which likely represent phosphorylated cryptochrome photoreceptors (Fig 1A and 1B; S1A Fig). This suggests that YFP-COP1 preferentially associates with phosphorylated cryptochromes in B-exposed seedlings.

To verify that our observation of B-dependent association of cryptochrome with COP1 is not specific to this particular YFP-COP1 line, we performed similar co-immunoprecipitation experiments with an independent *35S*::*YFP-COP1* line described previously [33]. Also using this line, only B-exposed seedlings showed a co-immunoprecipitation of CRY1 and CRY2 by YFP-COP1 (S1B and S1C Fig), further confirming that cryptochromes associate with COP1 only after B-exposure *in planta*.

### SPA proteins are required for the B-induced association between COP1 and CRY1 *in vivo*, while CRY2 interacts with COP1 independently of SPA proteins

**Fig 2:**
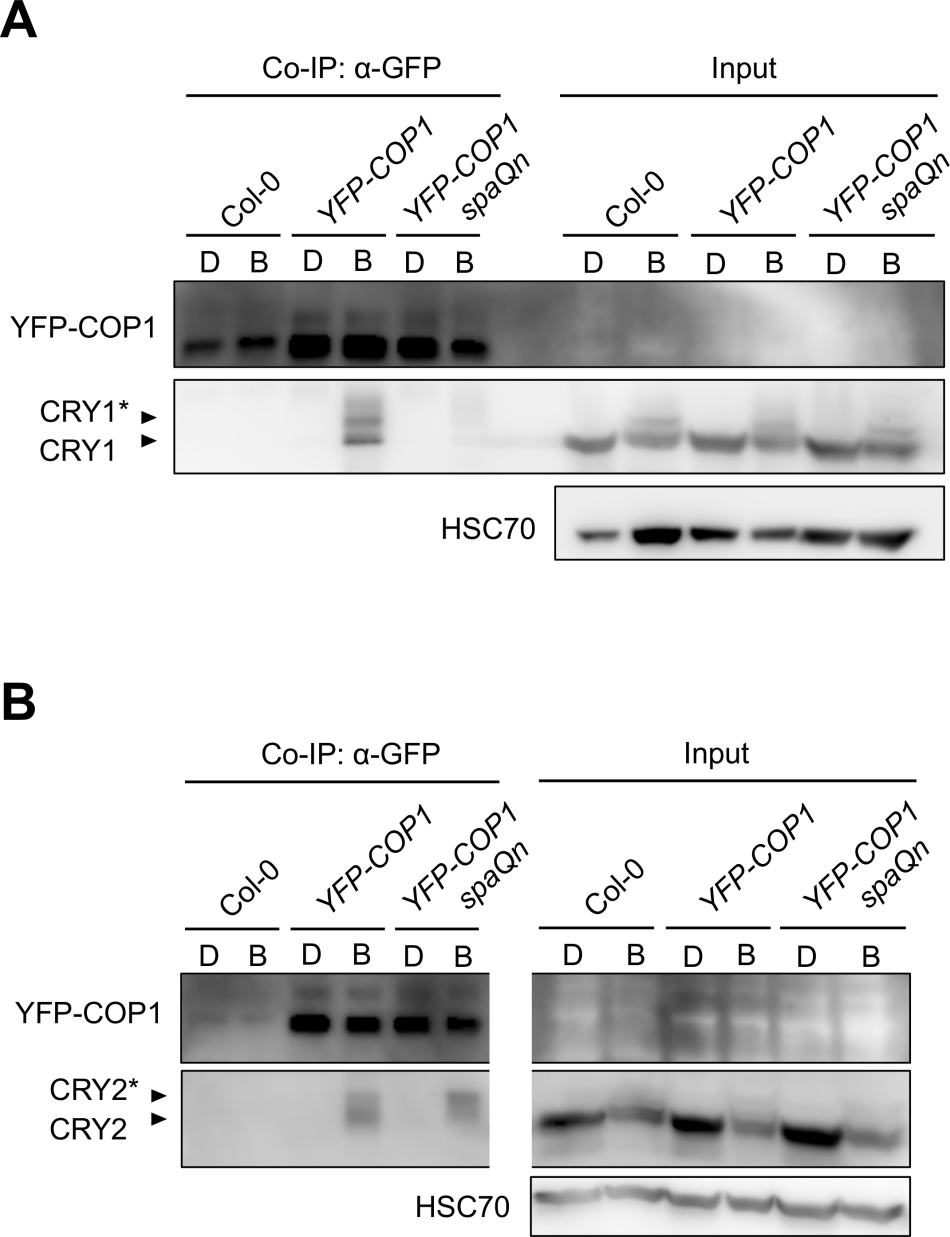
The B-induced *in vivo*-association of COP1 with CRY1 requires SPA proteins, while COP1 associates with CRY2 independently of SPA. (**A**, **B**) Co-immunoprecipitation of CRY1 (**A**) and CRY2 (**B**) by YFP-COP1 in a *SPA* wild-type (*YFP-COP1*) or *spa* null background (*YFP-COP1 spaQn*). Seedlings were grown in darkness for 4 days (D) and subsequently transferred to blue light (B) of a fluence rate of 50 μmol m^-2^ s^-1^ for 1 h (**A**) or 5 min (**B**). Protein extracts were immunoprecipitated using α-GFP beads. YFP-COP1 was detected using α-GFP antibodies. YFP-COP1 expression is very low and frequently only detectable after immunoprecipitation. CRY1 and CRY2 were detected using α-CRY1 and α-CRY2 antibodies. Asterisks likely indicate phosphorylated CRY1 and CRY2, respectively. The samples shown in (**B**) were on the same membrane with one lane digitally removed in the center.

The B-induced association of COP1 with CRY1 and CRY2 *in planta* might involve a direct recognition of COP1 by photo-activated cryptochromes or an indirect effect mediated by SPA proteins, e.g. by serving as bridging proteins. The latter possibility seems plausible since SPA proteins have been shown to interact with CRY1 and CRY2 in a B-dependent fashion [14–16]. To investigate this possibility we asked whether cryptochromes associate with COP1 in the absence of SPA proteins, i.e. in a *spaQn* null mutant background that lacks all four SPA proteins [19]. To this end, we crossed the *35S*::*YFP-COP1* transgene into the *spaQn* mutant background and performed co-immunoprecipitation experiments. YFP-COP1 did not co-immunoprecipitate significant amounts of CRY1 in a *spaQn* mutant background, neither in dark-grown nor in B-exposed seedlings. In contrast, B-exposed *35S*::*YFP-COP1* seedlings that are wild-type for *SPA1-SPA4* clearly showed co-immunoprecipitation of CRY1 by YFP-COP1 (Fig 2A). These results indicate that SPA proteins are required for the COP1-CRY1 association in B.

Using the same genotypes, we performed co-immunoprecipitations for CRY2. We found that COP1 associated with CRY2 in the *spaQn* mutant background, i.e. also in the absence of SPA proteins (Fig 2B). B-dependency of the CRY2-COP1 complex formation was maintained in *spaQn* since CRY2 was not co-immunoprecipitated by YFP-COP1 in dark-grown seedlings (Fig 2B). Taken together, these results suggest that the mechanisms by which CRY1 and CRY2 interact with COP1/SPA in B are distinct, with CRY1 requiring SPA proteins while CRY2 can associate with COP1 even in the absence of SPAs. For both CRY1 and CRY2, SPA proteins are not required to prevent a cry-COP1 complex formation in darkness.

### The COP1/CRY1 interaction is enhanced by the presence of SPA1 in yeast cells

**Fig 3:**
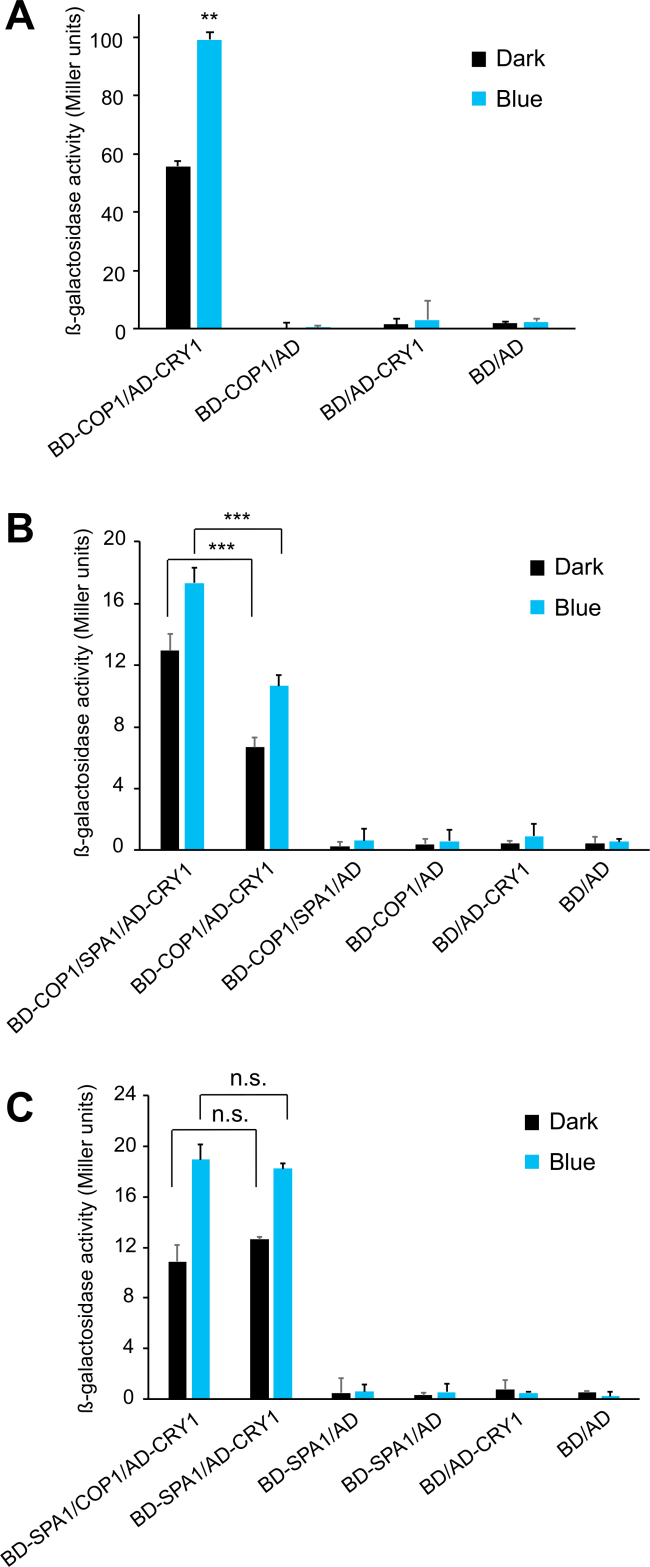
Co-expression of SPA1 increases the interaction between COP1 and CRY1 in yeast three-hybrid experiments. **(A)** Yeast two-hybrid assay analyzing the interaction between CRY1 and COP1. (**B**) Yeast three-hybrid assay analyzing the CRY1-COP1 interaction in the presence or absence of co-expressed SPA1. (**C**) Yeast three-hybrid assay analyzing the CRY1-SPA1 interaction in the presence or absence of co-expressed COP1. Co-transformed yeast cells were grown in darkness for 24 h and exposed to B (50 μmol m^-2^ s^-1^) or kept in darkness for 24 h before measuring ß-galactosidase activity. Error bars represent the SEM of three biological replicates. Asterisks indicate significant differences between the indicated comparisons (** *p* < 0.01, *** *p* < 0.001, n.s. not significant at *p* = 0.05).

To investigate whether SPA1 serves as a bridging protein to allow a B-dependent interaction between CRY1 and COP1, we examined these interactions in yeast whose genome contains neither *COP1-* nor *SPA*-homologous genes. We first tested the direct interaction between COP1 and CRY1 using the yeast two-hybrid system. As reported previously [30], we found that CRY1 directly interacted with COP1 in yeast. This interaction was enhanced when the co-transformed yeast cells were exposed to B when compared to cells kept in darkness (Fig 3A). We subsequently switched to the yeast three-hybrid system and compared the interaction strength between COP1 and CRY1 in the presence or absence of SPA1. Co-expression of SPA1 enhanced the COP1-CRY1 interaction almost 2-fold, both in darkness and in B (Fig 3B). This result indicates that SPA1 increases the affinity of CRY1 for COP1 and, therefore, suggests that the COP1/SPA complex has a higher affinity for CRY1 than COP1 alone which is in agreement with our findings from *in planta* co-immunoprecipitation experiments.

The promotive function of SPA1 in yeast cells was independent of light, i.e. equally observed in B and in darkness. This suggests that yeast does not fully mimic the *in planta* scenario of protein interactions. We speculate that also in dark-grown yeast, CRY1 may at least partially be in a conformation that resembles active CRY1, i.e. exposes the COP1/SPA-interacting domain(s) of CRY1. This may also explain why others found a constitutive interaction between CRY1 and COP1 in yeast [30]. Consistent with this idea, also SPA1 interacted with CRY1 in dark-grown yeast cells and this basal-level interaction was enhanced by B (S2 Fig). This is in contrast to previous results showing a fully B-dependent interaction of SPA1 with CRY1 in yeast, however using different yeast two-hybrid fusion constructs [15, 16].

We subsequently considered the reverse possibility that COP1 is required for the B-induced association of SPA1 with CRY1. Using the yeast three-hybrid system, we found that co-expression of COP1 as a bridge protein did not affect the SPA1-CRY1 interaction, neither in darkness nor in blue-light (Fig 3C).

### A SPA1 deletion-protein lacking the COP1-interacting coil-coil domain fails to associate with CRY1 in B

**Fig 4:**
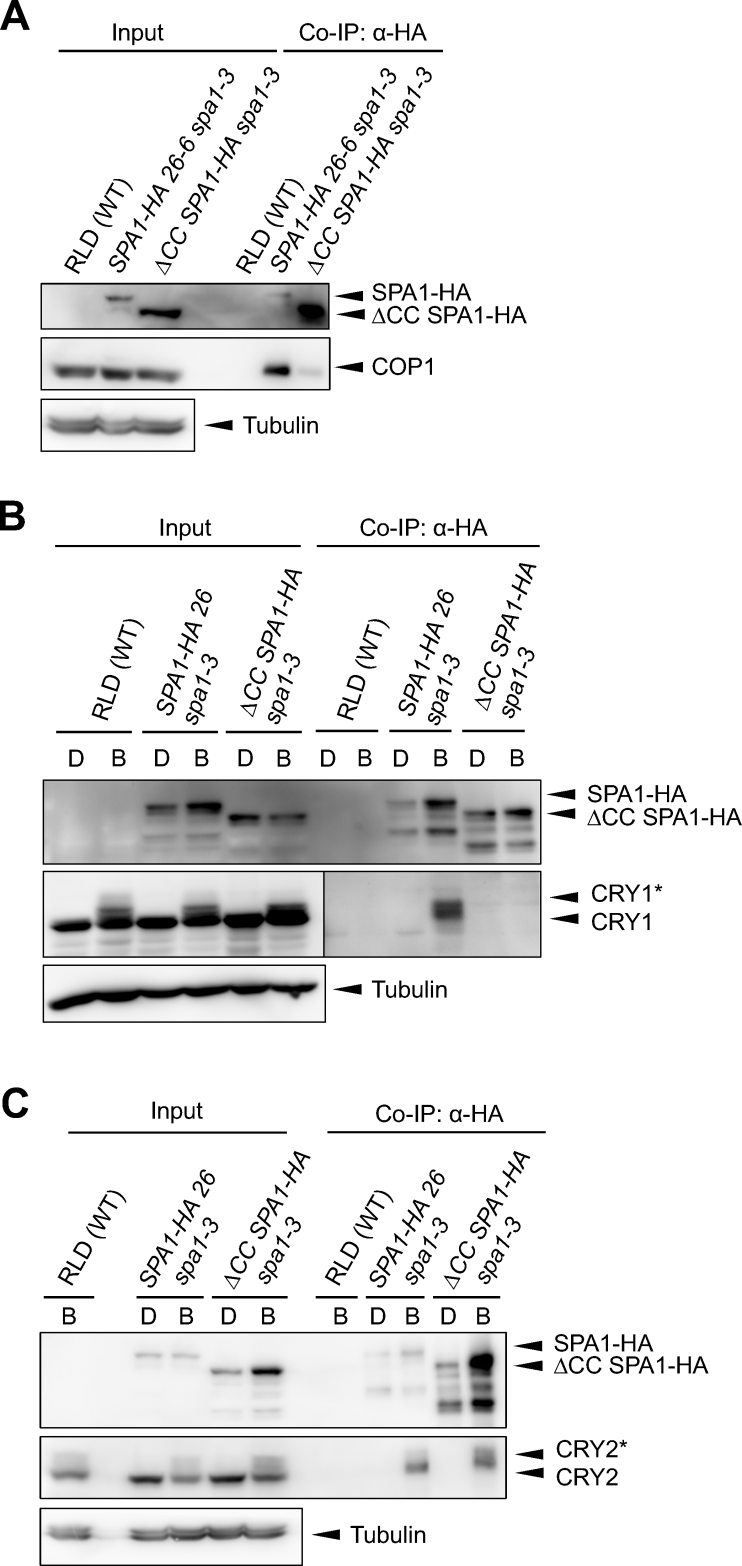
A SPA1 deletion-protein defective in COP1-interaction shows no or a strongly reduced *in vivo*-association with CRY1 and CRY2, respectively. (**A**) ΔCC SPA1-HA lacking the coiled-coil domain fails to interact with COP1 *in vivo*. Seedlings were grown in darkness for 4 d. **(B, C)** Co-immunoprecipitation of CRY1 (**B**) and CRY2 (**C**) by SPA1-HA and ΔCC SPA1-HA. Seedlings were grown in darkness for 4 days (D) and subsequently transferred to blue light (B) of a fluence rate of 50 μmol m^-2^ s^-1^ for 1 h (**B**) or 5 min (**C**). HA-tagged proteins were immunoprecipitated using α-HA-coupled beads. SPA1-HA and ΔCC SPA1-HA were detected using α-HA antibodies. CRY1 and CRY2 were detected using α-CRY1 and α-CRY2 antibodies. Asterisks likely indicate phosphorylated CRY1 and CRY2, respectively. Images in (**B**) separated by a vertical bar represent the same membrane which was exposed for different periods of time.

The yeast three-hybrid system may not fully reflect the *in planta* action of COP1/SPA and cryptochromes. We therefore addressed the question whether COP1 is required for the cryptochrome-SPA1 interaction using Arabidopsis seedlings. Since *cop1* null mutants barely germinate and show arrested growth at the very early seedling stage, we could not obtain enough tissue to analyze the SPA1-CRY1 interaction in a *cop1*-null mutant background using *in vivo* co-immunoprecipitation. We therefore applied an indirect approach and asked whether a SPA1 deletion-derivative lacking the COP1-interacting coiled-coil domain (*SPA1*:Δ*CC SPA1-HA*) [34, 35] can associate with CRY1 and CRY2. We first confirmed thatΔCC SPA1-HA did not interact with COP1 *in vivo*. Indeed, as expected, ΔCC SPA1-HA failed to associate with COP1, as COP1 was not co-immunoprecipitated by ΔCC SPA1-HA in transgenic ΔCC SPA1-HA-expressing seedlings. In contrast, full-length SPA1-HA strongly interacted with COP1 (Fig 4A). We therefore conclude that the ΔCC SPA1-HA protein is not part of a COP1/SPA complex and thus allows us to test whether ΔCC SPA1 is capable of interacting with CRY1 *in vivo*, i.e. in the absence of COP1. It was shown previously that the coiled-coil domain of SPA1 is not involved in the SPA1-CRY1 and SPA1-CRY2 interactions [14–16]. Hence, ΔCC SPA1 should not be affected in a CRY association *per se*.

We found that ΔCC SPA1 did not associate with CRY1 *in vivo* (Fig 4B). This result shows that the coiled-coil domain in SPA1 is needed for the association of SPA1 with CRY1. To re-confirm that the coiled-coil domain is not necessary for the SPA1-CRY1 interaction *per se*, we performed a yeast two-hybrid assay with the identical ΔCC SPA1 deletion protein that we used for the *in vivo* co-immunoprecipitation. Indeed, ΔCC SPA1 was not impaired in the interaction with CRY1 in the yeast two-hybrid assay, as shown previously [15, 16]; it interacted even more strongly with CRY1 than full-length SPA1. B-exposure also enhanced the interaction to a similar extent for ΔCC SPA1-CRY1 and full-length SPA1-CRY1 (S2 Fig). Taken together, these results indicate that only *in planta Δ*CC SPA1 fails to associate with CRY1 in B.

We subsequently asked whether the coiled-coil domain of SPA1 is also required for the interaction of SPA1 with CRY2. Fig 4C shows that CRY2 is co-immunoprecipitated by ΔCC SPA1-HA in B. However, the affinity is strongly reduced when compared to full-length SPA1, represented by a lower amount of co-immunoprecipitated CRY2 protein when compared to the full-length SPA1-expressing line (Fig 4C). We therefore conclude that ΔCC SPA1 is capable of interacting with CRY2, though with much reduced affinity.

## Discussion

Exposure of Arabidopsis plants to B activates the cryptochrome photoreceptors CRY1 and CRY2 which subsequently initiate light signaling by inhibiting the COP1/SPA E3 ubiquitin ligase. It was shown previously that both COP1 and SPA proteins can directly interact with cryptochromes. While the interaction between cryptochromes and SPA1 is B-dependent [14–16], it was proposed that CRY1 interacts with COP1 constitutively [30]. This suggested that cryptochromes might be in a complex with COP1 even in darkness, though in a non-functional fashion [12]. Here, we have shown by *in vivo* co-immunoprecipitation that COP1 associates with CRY1 and CRY2 *in vivo* only after seedlings were exposed to B; no complex formation between COP1 and CRY1 or CRY2 was observed in dark-grown seedlings. Hence, our results suggest that only photo-activated cryptochromes recognize COP1. These results further suggest that exposure to B allows cryptochromes to bind not only to SPA1 but to the COP1/SPA complex. This is consistent with previous, independent results showing that COP1 associates with CRY2 in B but not in red light [14]. Moreover, since SPA1 is in a complex with COP1 in darkness [34–36] and fails to associate with CRY1 and CRY2 in darkness *in vivo* [14–16], it is conclusive that COP1, like SPA1, does not associate with the cryptochromes in dark-grown seedlings. We found that COP1 preferentially interacts with the phosphorylated, higher-mobility isoforms of CRY1 and CRY2 that are formed upon B-irradiation. A similar observation was made for the B-dependent association of SPA1 and SPA2 with the higher-mobility isoforms of cryptochromes [26, 37]. The B-induced phosphorylation of cryptochromes is a key step in cryptochrome activation and function [5]. It is therefore consistent that the COP1/SPA complex primarily recognizes the active conformation of CRY1 and CRY2. It was hypothesized that phosphorylation of CRY2 causes a conformational change in the photoreceptor from a "closed" to an "open" conformation in which the CCE domain becomes exposed and accessible for interacting proteins [9]. Our results are consistent with this hypothesis.

We considered two not mutually exclusive scenarios to explain the B-dependent associations of CRY1 and CRY2 with COP1. They might reflect an intrinsic ability of COP1 to recognize photoactivated cryptochromes and/or they might require the activity of a third protein, such as SPA1. The latter possibility seems plausible since SPA proteins have been shown to interact with CRY1 and CRY2 in a B-dependent fashion [14–16]. Our finding that the B-induced CRY1-COP1 complex formation is not observed in a *spaQn* mutant background indicates that CRY1 and COP1 are not sufficient for light-responsiveness of this interaction *in vivo*. Hence, SPA proteins are indeed required for the B-induced association of CRY1 with COP1. Consistent with this result, co-expression of SPA1 enhanced the interaction of CRY1 with COP1 in the yeast three-hybrid assay. On the other hand, in the yeast two-hybrid assay, the ability of COP1 to bind CRY1 was slightly, about 2-fold, higher in B when compared to dark-grown yeast. This suggests that COP1, like SPA1, has at least some intrinsic ability to preferentially recognize photoactivated CRY1 as it is produced in yeast. Since both SPA1 and COP1 bind the same domain of CRY1, the CCE domain, with their respective, highly homologous WD-repeat domains [15, 16, 29, 30], it would be plausible that both SPA1 and COP1 have a higher affinity for photoactivated CRY1. Nevertheless, as shown previously [30], we found a basal interaction between COP1 and CRY1 in dark-grown yeast and in the absence of SPA1. This is in contrast to our observations derived from *in vivo* co-immunoprecipitations. Thus, the yeast system does not fully mimic the *in planta* protein interactions, possibly because it lacks regulatory mechanisms found only in plants.

SPA1 might be required for the B-induced CRY1-COP1 association *in vivo* because it serves as a bridging protein that subsequently enhances a direct interaction between CRY1 and COP1. Alternatively, the structure of the COP1/SPA complex, as it is formed in darkness, might have a higher affinity for photoactivated CRY1 than either SPA or COP1 alone. Our finding that co-expression of COP1 did not increase the CRY1-SPA1 interaction in the yeast three-hybrid assay argues against the latter possibility. However, *in vivo*, a SPA1 deletion-derivative that does not bind COP1 did not interact with CRY1 in B. This suggests that SPA1 can only bind photoactivated CRY1 when bound to COP1 *in vivo*, i.e. that SPA1 and COP1 reciprocally require each other for a strong interaction with CRY1 in B. Hence, SPA1 and COP1 may cooperatively bind CRY1 in B. However, since the deletion of the coiled-coil domain of SPA1 does not only abolish the heterodimerization of SPA1 with COP1 but also strongly reduces SPA1 homodimerization [36], we cannot exclude the possibility that ΔCC SPA1 fails to interact with CRY1 because it is a monomer rather than a dimer. Further experiments are necessary to unravel whether there is indeed reciprocal cooperativity between SPA1 and COP1 in the interaction with CRY1 *in vivo*.

In contrast to the B-induced CRY1-COP1 interaction which was dependent on SPA proteins *in vivo*, CRY2 formed a complex with COP1 also in the absence of SPA proteins. This suggests that the CRY2-COP1 interaction differs from the CRY1-COP1 interaction. In yeast and plants, it was reported previously that over-expression of SPA1 enhanced the CRY2-COP1 interaction in B [14]. We therefore consider it possible that SPA proteins also increase the CRY2-COP1 interaction but to a much lesser extent that is not detectable in our co-immunoprecipitation assays expressing all four SPA proteins at native levels. Alternatively, there may be distinct activities and interactions among the four SPA proteins in the heterologous COP1/SPA complexes. The stronger requirement of the four SPA proteins for the CRY1-COP1 interaction than for the CRY2-COP1 interaction *in vivo* might relate to the sequence divergence of the respective CCE domains in CRY1 and CRY2. Both, CCE1 and CCE2 of CRY1 and CRY2, respectively, are responsible for the interaction with the WD-repeat domain of COP1, but the sequence conservation between CCE1 and CCE2 is rather low when compared to the high sequence conservation between the PHR domains of CRY1 and CRY2 [5]. Also, the interactions of CRY1 and CRY2 with the SPA proteins profoundly differ: the N-terminal kinase-like domain of SPA1 interacts with the PHR domain of CRY2, while the C-terminal WD-repeat domain of SPA1 interacts with the CCE domain of CRY1 [14–16]. These differential interacting domains may also play a role in the respective requirement of SPA proteins for the B-induced interaction of COP1 with CRY1 and CRY2, respectively. We propose that a B-induced interaction of COP1 with the CCE domain of photoactivated CRY2 is complemented by an additional interaction platform between the N-terminal domain of SPA and the N-terminal domain of CRY2.

## Materials and methods

### Plant material and growth conditions

The transgenic *35S*::*YFP-COP1* lines were described previously [32, 33]. The *35S*::*YFP-COP1 spaQn* line was described in [31], the transgenic *SPA1*::*SPA1-HA 26 spa1-3* and *SPA1*::Δ*CC-SPA1-HA spa1-3* lines were described in [38].

LED light sources and seedling growth conditions were as described previously [20, 39].

### *In vivo* co-immunoprecipitation

These experiments were performed as described in [37]. Briefly, seedlings were homogenized in extraction buffer (50 mM Tris pH 7.5, 150 mM NaCl, 1 mM EDTA, 10% glycerol, 0.1% Triton X-100, 5 mM DTT, 1% protease inhibitor cocktail (Sigma-Aldrich), 10 μM MG132, phosSTOP phosphatase inhibitor cocktail (Roche, 1 tablet/5 ml buffer)). After centrifugation, 1.5–3 mg of total protein of the supernatant was subjected to co-immunoprecipitation using the magnetic μMACS Anti-HA isolation kit (Miltenyi Biotec) for HA-tagged bait proteins and magnetic μMACS Anti-GFP isolation kit (Miltenyi Biotec) for YFP-tagged bait proteins according to the manufacturer´s instructions. Twice as much total protein was added for *YFP-COP1 spaQn* seedlings than for *YFP-COP1* seedlings. Proteins were detected by Western blot using antibodies against GFP (Roche Diagnostics, Mannheim, Germany), HA (Roche Diagnostics, Mannheim, Germany), HSC70 (Stressgen Biotechnologies, San Diego, USA), α-tubulin (Sigma-Aldrich, Munich, Germany), CRY1 [40] and CRY2 [41]. All experiments were repeated 3–6 times with similar results, except for the experiments shown in Fig 4A and S1 Fig which were repeated twice with similar results. A representative image is shown for each experiment.

### Yeast two-hybrid and three-hybrid experiments

For yeast two-hybrid assays to study the interaction between COP1 and CRY1, the bait vector pEG202 expressing the LexA DNA binding domain fused to COP1 and the prey vector pB42AD expressing the B42 transcriptional activation domain fused to CRY1 were used. pEG202 expressing ΔCC-SPA1 [38] or full-length SPA1 was utilized to study the interaction between CRY1 and SPA1 lacking the coiled-coil domain or full-length SPA1. Empty vectors were used as negative controls. The plasmids used in this study are listed in S1 Table; their construction is described below. The respective plasmid pairs were co-transformed into the yeast strain EGY48 (p8op-LacZ) (Clontech) following the Yeast Protocol Handbook (Clontech) and selected on synthetic drop-out auxotrophic plates (SD -H, -W, -Ura, +Glucose). Three to 4 transformed yeast colonies were then plated on induction media (IM -H, -W, -Ura, +Galactose, +Raffinose) and grown either in darkness for 48 h or grown in darkness for 24 h and transferred to B (50 μmol m^-2^s^-1^) for another 24 h. The cells were then scraped from the plate (dark samples under red light and B samples under B), OD_600_ was adjusted to 1.00 to have an equal number of cells in all samples and ß-galactosidase activity was measured using ortho-Nitrophenyl-β-galactoside (ONPG) as a substrate with three biological and two technical replicates each. Miller Units corresponding to the ß-galactosidase activity were calculated for each sample according to the yeast protocol handbook (Clontech).

For yeast three-hybrid assays, two separate sets of experiments were performed with SPA1 and COP1 as bridge proteins, respectively. In the first experiment with SPA1 as bridge protein, the interaction between COP1 and CRY1 was measured with and without the co-expression of SPA1. In the second experiment, the interaction between SPA1 and CRY1 was measured with and without the co-expression of COP1. The vector pBridge-GW was used which expresses the GAL4 DNA-binding domain fused to the bait protein (COP1 or SPA1, respectively) and a bridge protein (SPA1 or COP1, respectively). The prey protein CRY1 was expressed as a fusion protein with the GAL4 activation domain in the vector pACT2-GW. The plasmids used in the study are listed in S1 Table; their construction is described below. The plasmid combinations were co-transformed into the yeast strain Y190 (Clontech) and selected on synthetic drop-out medium (SD -L, -W). Three to 4 transformed yeast colonies were then sub-cultured on induction medium (IM -L, -W, -M) to express the respective bridge protein. Since the methionine-repressible promoter in pBridge-GW is leaky and, moreover, the lack of methionine influences yeast growth, pBridge-GW constructs lacking the coding sequence for the respective bridge protein were used as controls and grown in the same induction medium without methionine. Further growth conditions, collection of yeast cells as well as the measurement of ß-galactosidase activity were performed as described above.

For an overview of all constructs used, please see S2 Table. For yeast two-hybrid assays, the bait constructs (pEG202_COP1, pEG202_SPA1 and pEG202_ΔCC-SPA1) were constructed by cloning the respective CDS into the pEG202 vector. To this end, the respective CDS was amplifyied using primers having NcoI and XhoI restriction sites (NcoI_SPA1/COP1_Forward, XhoI_SPA1/COP1_Reverse, respectively) followed by ligation into pEG202 that had been digested with the same enzymes. The ΔCC-SPA1 CDS was amplified from ΔCC-SPA1-3xHA pBS [38] which produces a deletion of the amino acids 566–639 of SPA1. Similarly, the prey construct (pB42AD_CRY1) was constructed by amplifying the CDS of CRY1 using the primers EcoRI-CRY1-F and XhoI-CRY1-R followed by ligation into the pB42AD vector using EcoRI and XhoI restriction sites.

For yeast three hybrid assays, the bait constructs (pBridge-GW_BD-COP1_bridge-empty and pBridge-GW_BD-SPA1_bridge-empty) were prepared by utilising restriction sites in an expanded Multiple Cloning Site of a pBridge-GW. For this purpose, COP1 and SPA1 CDSs were amplified with primers containing NheI/ApaI and SacII/NheI restriction sites, respectively (COP1_NheI_FP and COP1_ApaI_RP; SPA1_SacII_FP and SPA1_Nhe1_RP), and ligated into the pBridge-GW plasmid that had been digested with the respective restriction enzymes. For expressing SPA1 and COP1 as bridge proteins, the entry clones COP1-pDONR221 and SPA1-pENTR3C were used for Gateway LR reactions which transferred the respective CDS into the Gateway sites of pBridge-GW_BD-COP1-bridge empty, pBridge-GW_BD-SPA1_bridge-empty and pBridge-GW to generate pBridge-GW_BD-COP1_bridge-SPA1, pBridge-GW_BD-empty_bridge-SPA1, pBridge-GW_BD-SPA1_bridge-COP1 and pBridge-GW_BD-empty_bridge-COP1. For expressing the AD-CRY1 fusion protein (pACT2-GW_CRY1), pDONR221 CRY1 was used as entry clone for a Gateway LR reaction which transferred the CRY1 CDS into pACT2-GW carrying a Gateway cassette in the vector pACT2. All inserts were sequenced for confirmation. The primers used for cloning are listed in S1 Table.

### Accession numbers

SPA1 (At2g46340), SPA2 (At4g11110), SPA3 (At3g15354), SPA4 (At1g53090), COP1 (At2g32950), cry1 (At4g08920), cry2 (At1g04400).

## Supporting information

S1 Fig COP1 associates with CRY1 and CRY2 in a blue-light dependent manner. Co-immunoprecipitation of CRY1 (**A, B**) and CRY2 (**C**) by YFP-COP1. Transgenic seedlings of two independent 35S::*YFP-COP1* lines (**A**: Oravecz et al., 2006; **B,C**: Subramanian et al., 2006) were grown in darkness (D) for 4 days and subsequently transferred to blue light (B) of a fluence rate of 50 μmol m^-2^ s^-1^ for 1 h (**A, B**) or 5 min (**C**). Protein extracts were immunoprecipitated using α-GFP beads. YFP-COP1 was detected using α-GFP antibodies; CRY1 and CRY2 were detected using α-CRY1 and α-CRY2 antibodies. Asterisks likely indicate phosphorylated CRY1 and CRY2, respectively. Images separated by a vertical bar represent the same membrane which was exposed for different periods of time.
(PDF)

S2 Fig SPA1 lacking the coiled-coil domain interacts with CRY1 in the yeast two-hybrid assay. Yeast two-hybrid assay with SPA1 or a SPA1 deletion-derivative lacking the coiled-coil domain (ΔCC SPA1) as baits and CRY1 as prey. Co-transformed yeast cells were grown in darkness for 24 h and exposed to B (50 μmol m^-2^ s^-1^) or kept in darkness for 24 h before measuring ß-galactosidase activity. Error bars represent the SEM of three biological replicates.
(PDF)

S1 Table List of primers used for cloning. (DOCX)

S2 Table Overview of plasmids used for yeast two-hybrid and three-hybrid experiments. (DOCX)
