# Supplementary material for: The blue light-induced interaction of cryptochrome 1 with COP1 requires SPA proteins during Arabidopsis light signaling
Source: PLoS Genet. 2017 Oct 9;13(10):e1007044. doi: 10.1371/journal.pgen.1007044 (PMC5648270; doi:10.1371/journal.pgen.1007044)
Supplement: S1 Fig — Co-immunoprecipitation of CRY1 (A, B) and CRY2 (C) by YFP-COP1. Transgenic seedlings of two independent 35S::YFP-COP1 lines (A: Oravecz et al., 2006; B,C: Subramanian et al., 2006) were grown in darkness (D) for 4 days and subsequently transferred to blue light (B) of a fluence rate of 50 μmol m-2 s-1 for 1 h (A, B) or 5 min (C). Protein extracts were immunoprecipitated using α-GFP beads. YFP-COP1 was detected using α-GFP antibodies; CRY1 and CRY2 were detected using α-CRY1 and α-CRY2 antibodies. Asterisks likely indicate phosphorylated CRY1 and CRY2, respectively. Images separated by a vertical bar represent the same membrane which was exposed for different periods of time. (PDF) [file pgen.1007044.s001.pdf]

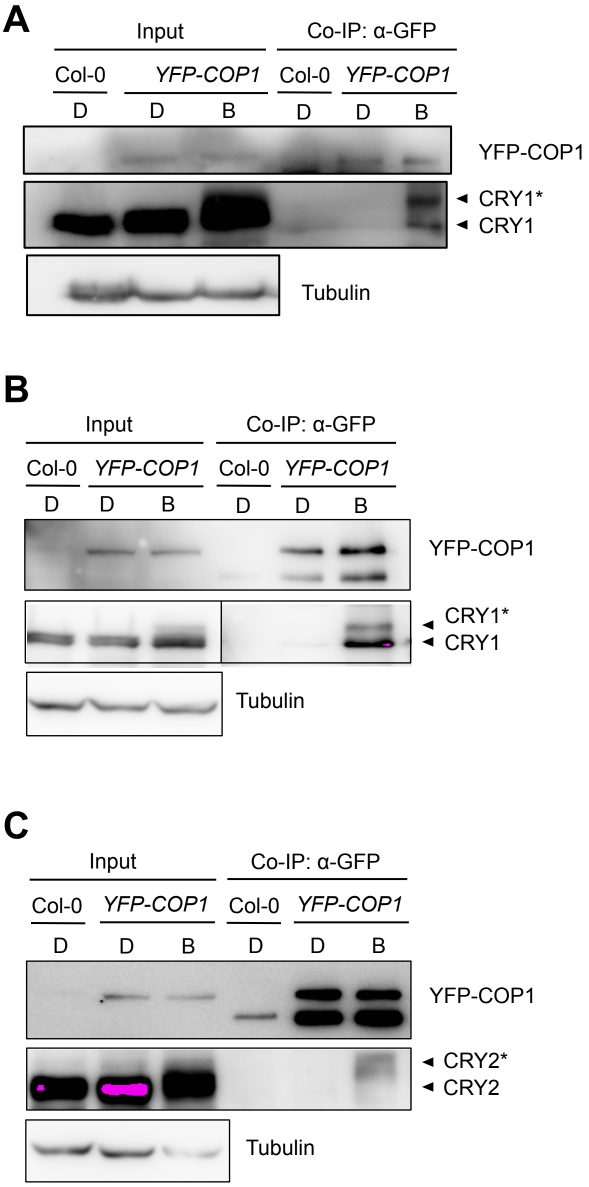

Figure S1: COP1 associates with CRY1 and CRY2 in a blue-light dependent manner

Co-immunoprecipitation of CRY1 (**A, B**) and CRY2 (**C**) by YFP-COP1.

Transgenic seedlings of two independent 35S::*YFP-COP1* lines (**A**: Oravecz et al., 2006; **B,C**: Subramanian et al., 2006) were grown in darkness (D) for 4 days and subsequently transferred to blue light (B) of a fluence rate of 50 µmol m^{-2} s^{-1} for 1 h (**A, B**) or 5 min (**C**). Protein extracts were immunoprecipitated using α-GFP beads. YFP-COP1 was detected using α-GFP antibodies; CRY1 and CRY2 were detected using α-CRY1 and α-CRY2 antibodies. Asterisks likely indicate phosphorylated CRY1 and CRY2, respectively. Images separated by a vertical bar represent the same membrane which was exposed for different periods of time.
