# Supplementary material for: The blue light-induced interaction of cryptochrome 1 with COP1 requires SPA proteins during Arabidopsis light signaling
Source: PLoS Genet. 2017 Oct 9;13(10):e1007044. doi: 10.1371/journal.pgen.1007044 (PMC5648270; doi:10.1371/journal.pgen.1007044)
Supplement: S2 Fig — Yeast two-hybrid assay with SPA1 or a SPA1 deletion-derivative lacking the coiled-coil domain (ΔCC SPA1) as baits and CRY1 as prey. Co-transformed yeast cells were grown in darkness for 24 h and exposed to B (50 μmol m-2 s-1) or kept in darkness for 24 h before measuring ß-galactosidase activity. Error bars represent the SEM of three biological replicates. (PDF) [file pgen.1007044.s002.pdf]

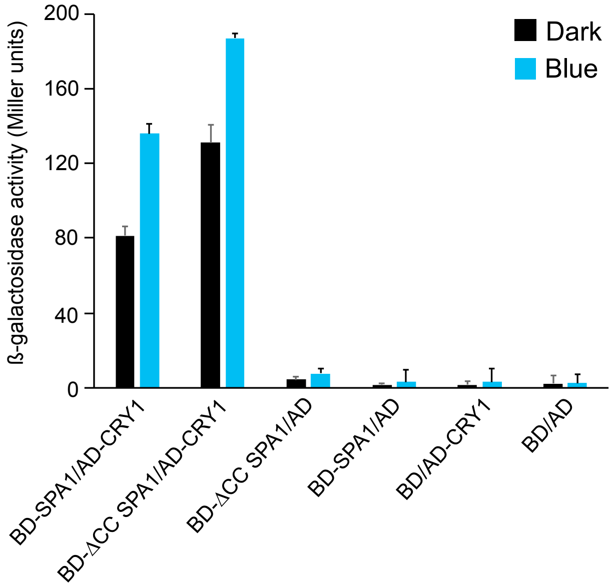


**Figure S2: SPA1 lacking the coiled-coil domain interacts with CRY1 in the yeast two-hybrid assay**

Yeast two-hybrid assay with SPA1 or a SPA1 deletion-derivative lacking the coiled-coil domain (ΔCC SPA1) as baits and CRY1 as prey. Co-transformed yeast cells were grown in darkness for 24 h and exposed to B (50 µmol $m^{-2}$ $s^{-1}$) or kept in darkness for 24 h before measuring ß-galactosidase activity. Error bars represent the SEM of three biological replicates.
