# Supplementary material for: The blue light-induced interaction of cryptochrome 1 with COP1 requires SPA proteins during Arabidopsis light signaling
Source: PLoS Genet. 2017 Oct 9;13(10):e1007044. doi: 10.1371/journal.pgen.1007044 (PMC5648270; doi:10.1371/journal.pgen.1007044)
Supplement: S1 Table — (DOCX) [file pgen.1007044.s003.docx]

**Table S1: List of primers used for cloning**

| Name of the primer | Sequence (5´ to 3´) |
| --- | --- |
| NcoI_SPA1_Forward | CATGccatggATGCCTGTTATGGAAAGAGTAG |
| XhoI_SPA1_Reverse | GGTCctcgagTCAAACAAGTTTTAGTAGCTTCA |
| NcoI_COP1_Forward | CCGAccatggATGGAAGAGATTTCGACGGATC |
| XhoI_COP1_Reverse | GGTCctcgagTCACGCAGCGAGTACCAGAACT |
| EcoRI-CRY1-F | CCGAgaattcATGTCTGGTTCTGTATCTGGTT |
| XhoI-CRY1-R | GGTCctcgagTTACCCGGTTTGTGAAAGCCGT |
| COP1_NheI_FP | TAAGCAgctagcATGGAAGAGATTTCGACG |
| COP1_ApaI_RP | TGCTTAgggcccTCACGCAGCGAGTACCAG |
| SPA1_SacII_FP | TAAGCAccgcggccATGCCTGTTATGGAAAGA |
| SPA1_Nhe1_RP | TGCTTAgctagcTCAAACAAGTTTTAGTAG |
