# Supplementary material for: The blue light-induced interaction of cryptochrome 1 with COP1 requires SPA proteins during Arabidopsis light signaling
Source: PLoS Genet. 2017 Oct 9;13(10):e1007044. doi: 10.1371/journal.pgen.1007044 (PMC5648270; doi:10.1371/journal.pgen.1007044)
Supplement: S2 Table — (DOCX) [file pgen.1007044.s004.docx]

**Table S2: Overview of plasmids used for yeast two-hybrid and three-hybrid experiments**

Plasmids used for yeast two-hybrid assays

| Name of the plasmid | BD | AD |
| --- | --- | --- |
| pEG202_COP1 | COP1 | - |
| pEG202_SPA1 | SPA1 | - |
| pEG202_∆CC-SPA1 | ∆CC-SPA1 | - |
| pB42AD_CRY1 | - | CRY1 |
| pEG202 | - | - |
| pB42AD | - | - |

Plasmids used for yeast three-hybrid assays

| Name of the plasmid | BD | Bridge | AD |
| --- | --- | --- | --- |
| pBridge-GW_BD-COP1_bridge-SPA1 | COP1 | SPA1 | - |
| pBridge-GW_BD-empty_bridge-SPA1 | - | SPA1 | - |
| pBridge-GW_BD-SPA1_bridge-COP1 | SPA1 | COP1 | - |
| pBridge-GW_BD-empty_bridge-COP1 | - | COP1 | - |
| pBridge-GW_BD-COP1_bridge-empty | COP1 | - | - |
| pBridge-GW_BD-SPA1_bridge-empty | SPA1 | - | - |
| pACT2-GW_CRY1 | - | - | CRY1 |
| pBridge-GW | - | - | - |
| pACT2-GW | - | - | - |
